# Carotenoids and Intestinal Harmony: Exploring the Link for Health †

**DOI:** 10.3390/foods13111599

**Published:** 2024-05-21

**Authors:** Helena R. Rocha, Manuela E. Pintado, Ana M. Gomes, Marta C. Coelho

**Affiliations:** CBQF—Centro de Biotecnologia e Química Fina—Laboratório Associado, Escola Superior de Biotecnologia, Universidade Católica Portuguesa, Rua Diogo Botelho 1327, 4169-005 Porto, Portugal; mhrocha@ucp.pt (H.R.R.); mpintado@ucp.pt (M.E.P.); amgomes@ucp.pt (A.M.G.)

**Keywords:** intestinal microbiota, natural pigments, modulation, fermentation, metabolites

## Abstract

Carotenoids, prominent lipid-soluble phytochemicals in the human diet, are responsible for vibrant colours in nature and play crucial roles in human health. While they are extensively studied for their antioxidant properties and contributions to vitamin A synthesis, their interactions with the intestinal microbiota (IM) remain poorly understood. In this study, beta (β)-carotene, lutein, lycopene, a mixture of these three pigments, and the alga *Osmundea pinnatifida* were submitted to simulated gastrointestinal digestion (GID) and evaluated on human faecal samples. The results showed varying effects on IM metabolic dynamics, organic acid production, and microbial composition. Carotenoid exposure influenced glucose metabolism and induced the production of organic acids, notably succinic and acetic acids, compared with the control. Microbial composition analysis revealed shifts in phyla abundance, particularly increased Pseudomonadota. The α-diversity indices demonstrated higher diversity in β-carotene and the pigments’ mixture samples, while the β-diversity analysis indicated significant dissimilarity between the control and the carotenoid sample groups. UPLC-qTOF MS analysis suggested dynamic changes in carotenoid compounds during simulated fermentation, with lutein exhibiting distinct mass ion fragmentation patterns. This comprehensive research enhances our understanding of carotenoid-IM interactions, shedding light on potential health implications and the need for tailored interventions for optimal outcomes.

## 1. Introduction

Carotenoids are a fascinating group of natural pigments widely distributed in nature and are responsible for the vibrant red, orange, and yellow hues [1] found in plants, bacteria, microalgae, and fungi [2,3]. These colourful lipophilic pigments [4,5] are commonly found in the plant kingdom as secondary metabolites, typically existing in the form of C-40-based tetraterpenoids [1]. However, in bacteria, there is a distribution of C-45- and C-50-based carotenoids as well [1].

In terms of their chemical composition, carotenoids can be divided into hydrocarbon carotenoids (including beta (β)-carotene and lycopene) and oxygenated carotenoids (xanthophylls) based on the presence or absence of oxygen atoms within their structures [6,7]. Carotenoids are exogenous nutrients for humans and animals [1], given their inability to synthesize them de novo [4,7]. Humans, in particular, exhibit a distinctive trait as indiscriminate accumulators, absorbing over 40 carotenoids from in their diet and accumulating them in the body without discrimination [1].

Carotenoids have diverse biological functions beyond their role in photosynthesis, contributing to plant energy conversion [1,8] and also as precursors to vitamin A, supporting vision [6], immune function [9], and cardiovascular health [10]. In addition, these pigments have antioxidant properties, neutralising free radicals, and potentially reduce the risk of chronic diseases [11,12].

The intestinal microbiota (IM), a diverse community of microorganisms comprising bacteria, viruses, and some eukaryotes, establishes itself in the digestive tract shortly after birth and remains present in the digestive tracts of humans and animals [13,14]. Although over 50 bacterial phyla have been identified, the human IM is primarily dominated by two, Bacteroidota and Bacillota, with Bacillota encompassing over 200 different genera, including *Lactobacillus* and *Clostridium*, and Bacteroidota primarily composed of dominant genera, such as *Bacteroides* and *Prevotella* [15]. Microbial composition varies along the gastrointestinal tract (GIT), with a dense population in the colon, hosting approximately 10^12^ bacterial cells per gram of intestinal content [13]. The IM performs essential functions, including protection against pathogens, immune system enhancement, production of antimicrobial substances, and control of epithelial cell proliferation and differentiation, playing a significant role in digestion, metabolism, and maintaining normal intestinal physiology and health [13,16,17,18].

Various factors, intrinsic and environmental, can influence the composition and function of the IM, including age, ethnicity, genetic markers, geographic area, lifestyle, diet, and drug intake [19,20]. Diet, in particular, plays a crucial role in modulating the IM, impacting nutrient processing, digestion, and immune cell growth and response [13]. The spatial confinement of the IM within the gastrointestinal lumen prompts the release of diverse metabolites, including bile acids, vitamins, tryptophan, and short-chain fatty acids (SCFAs), which exert profound effects on the host’s organs, regulating immune responses, nutrient absorption, host metabolism, and overall IM composition [21,22,23]. This intricate interplay between the IM and the host’s physiological functions is essential for maintaining health or contributing to the development of diseases [24].

However, there is a significant research gap in understanding carotenoid metabolism in the colon and its interactions with the IM, with limited information and clear evidence on this intriguing topic regarding isolated compounds, mixtures, and complex matrices such as algae.

Therefore, the present work aims to better understand the possible interaction between carotenoids and the IM. To accomplish this, three pure carotenoids ((β)-carotene, lutein and lycopene), a mixture of these three pigments, and the alga *Osmundea pinnatifida* were subjected to an in vitro simulation of gastrointestinal digestion. After characterization of the impact of this process on each tested condition, the digested carotenoids were evaluated on fresh human faecal samples to assess the effect on the IM’s metabolic and population dynamics.

## 2. Materials and Methods

### 2.1. In Vitro Gastrointestinal Digestion Simulation (GID)

#### 2.1.1. *Osmundea pinnatifida* Carotenoids’ Analysis

*Osmundea pinnatifida* is a red marine alga known for its diverse carotenoid composition and potential health benefits [25]. Table 1 presents the carotenoid composition of this alga, which was chosen as the matrix in this study to evaluate the bioaccessibility of these natural pigments. Carotenoid extraction followed a modified protocol based on Wright et al. [26], using a solvent mixture of water (10%) and acetone (90%).

#### 2.1.2. Sample Preparation

To ensure the stability of the carotenoid samples during digestion, a solution containing 1 mL of polysorbate 20 M (Tween 20) and 19 mL of phosphate-buffered solution (PBS) at pH 7.4 was incorporated into all the samples as a protective measure against their rapid degradation, addressing the lipophilic nature of these pigments. Specifically, the β-carotene solution comprised 11.9 mg of this carotene, 19 mL of PBS at pH 7.4, and 1 mL of Tween 20 (concentration: 0.57 mg/mL). The lutein and lycopene solutions were prepared similarly, and each contained 500 μL of the respective carotenoid (lutein concentration: 1.00 mg/mL; lycopene concentration: 2.50 mg/mL) and 2.5 mL of the Tween 20 solution. The mixed solution of β-carotene, lutein, and lycopene was prepared with 500 μL of each carotenoid from the previously prepared solutions and 1.5 mL of the Tween 20 solution (concentration: 6.00 mg/mL). In the case of *Osmundea pinnatifida* (Praia da Agudela, Portugal), the sample was homogenized by grinding the alga using a kitchen robot (Bimby TM6 Vorkwerk) and subsequently suspending 3 g of the alga in 3 mL of the Tween 20 solution (carotenoid concentration in the suspension: 93.48 mg/L).

#### 2.1.3. In Vitro Gastrointestinal Digestion (GID) Simulation

Simulated complete digestion of the carotenoid solutions was performed according to the standardized static digestion model INFOGEST 2.0 protocol described by Brodkorb et al. [27]. The procedure consists of simulation of the sequential phases that happen along the GIT. For each sample, one replica was performed and a negative control without any carotenoid sample (PBS) was used. First, to simulate the oral phase, 3 mL of a carotenoid solution (previously prepared) was mixed with 2.4 mL of Simulated Salivary Fluid (SSF) containing 0.015 mL of CaCl_2_ and 0.540 mL of α-amylase from human saliva (84 U/mg of powder; A1031-5KU; Sigma-Aldrich, St. Louis, MO, USA); the mixture was incubated in an orbital (Orbital Shaker MaxQ 6000) for 2 min at 37 °C at 200 rpm. Then, 4 mL of Simulated Gastric Fluid (SGF), containing 0.00250 mL of CaCl_2_ and 0.40 mL of RGE (resuspended rabbit gastric extract lipase and pepsin at 15 U/mg of powder; Lipolytech, Marseille, France), was added, and the resulting mixture was incubated in the above-mentioned orbital for 2 h at 37 °C at 130 rpm to simulate the gastric phase. The intestinal phase was simulated by adding 4 mL of Simulated Intestinal Fluid (SIF), containing 0.021 mL of CaCl_2_, 1.57 mL of bile salts (0.2 g/mL; bile extract porcine-B8631; Sigma-Aldrich, St. Louis, MO, USA) and 2.625 mL of pancreatin from porcine pancreas (6 U/mg of powder; P7545; Sigma-Aldrich, St. Louis, MO, USA), and then incubating this in the orbital for 2 h and 30 min at 37 °C at 45 rpm. To understand the possible absorption of the carotenoids in the samples upon digestion, a dialysis process was performed with 3.5 kDa membranes (pre-wetted RC Tubbing, Spectra/Por^®^6 Dialysis Membrane; 734–0652; VWR Chemicals, Radnor, PN, USA). The final content of the simulated digestion process was placed inside the membrane and a significant volume of water was used on the outside (enough to cover all sample that was inside the membrane). The volumes inside and outside the membrane were measured. The experimental setups were placed in the orbital overnight at 37 °C at 50 rpm to mimic the peristaltic movements. At the end of the process, the proportion that was found outside (OUT) of the dialysis membrane represented the sample fraction that was available for absorption and the proportion that was left inside (IN) of the dialysis membrane represented the non-absorbable fraction. During the gastrointestinal digestion simulation, 1 mL aliquots were collected for further analysis at the end of each step: mouth, stomach, small intestine, colon, and basolateral fraction.

### 2.2. In Vitro Fermentations

#### 2.2.1. Collection and Preparation of Faecal Inoculant

A pool of fresh faecal samples was provided by 5 healthy unrelated volunteer donors (3 men and 2 women with ages between 23 and 39 years old meeting the criterion of ≥18 and <50 years old) within the premises of Escola Superior de Biotecnologia (Porto, Portugal) according to the internal established protocol previously validated by the Health Ethics Committee from Universidade Católica Portuguesa (project n° 167). The volunteers had no chronic diseases nor allergies, as well as no recent consumption of probiotics or prebiotics (whether from food or supplements) in the preceding six months, or antibiotic usage during the same period. Each participant signed an informed consent form that provided detailed information about the study, in accordance with the Declaration of Helsinki conduct. The faecal samples were collected into sterile vials and were maintained under anaerobic conditions for a maximum of 2 h before use. The faecal inoculum was prepared by diluting the faecal matter (100 g/L) in Reduced Physiological Salt (RPS) solution (0.5 g/L cysteine hydrochloride-Merck, Darmstadt, Germany) and 8.5 g/L NaCl (LabChem, Zelienople, PN, USA) with a final pH value of 6.8. This was performed inside an anaerobic workstation (Don Whitley Scientific, West Yorkshire, UK), where the atmosphere consisted of 10% CO_2_, 5% H_2_, and 85% N_2_. The mixture was prepared and homogenized using a stomacher (Serward, Worthing, UK) for 2 min at 460 paddle beats per min [28,29,30,31,32].

#### 2.2.2. Fermentation Medium Preparation

The fermentation medium was composed of the following components: 5.0 g/L of tryptone soya broth (TSB) without dextrose (Fluka Analytical, St. Louis, MO, USA), 5.0 g/L bactopeptone (Becton Dickinson Biosciences, Franklin Lakes, NJ, USA), 5.0 g/L yeast nitrogen base, 0.5 g/L cysteine hydrochloride (Merck, Darmstadt, Germany), 1.0% (*v*/*v*) of salt solution A [consisting of 100.0 g/L NH_4_Cl (Merck, Darmstadt, Germany), 10.0 g/L MgCl_2_·6H_2_O (Merck, Darmstadt, Germany), and 10.0 g/L CaCl_2_·2H_2_O (Carlo Erba, Chaussée du Vexin, France)], 0.2% (*v*/*v*) of salt solution B [comprising 200.0 g/L K_2_HPO_4_·3H_2_O (Merck, Darmstadt, Germany], 0.2% (*v*/*v*) of a 0.5 g/L resazurin solution (Sigma-Aldrich Chemistry, St. Louis, MO, USA), and 1% (*v*/*v*) of a 10 mL/L trace mineral solution (ATCC, Manassas, VA, USA). The pH of the medium was adjusted to 6.8 and nitrogen gas was bubbled through it. As a positive control, 1 g of fructooligosaccharides at 2% (*m*/*v*) (FOS; Nutripar, Matosinhos, Portugal) was added, and the digested carotenoid from the colon fraction samples were added to the respective fermentation flasks (50 mL working volume) at a final concentration of 2%. The filled flasks were then sealed and subjected to autoclaving [28,29,30,31,32].

#### 2.2.3. Faecal Fermentations

The sterilized fermentation flasks, prepared as described in the Section 2.2.2, were aseptically inoculated with faecal inoculate at a concentration of 2% (*v*/*v*) and then incubated for 48 h at 37 °C within an anaerobic environment composed of 10% CO_2_, 5% H_2_, and 85% N_2_. Three samples were collected for each tested group at intervals of 0, 6, 12, 24, and 48 h during the incubation period, and the pH values were measured at each time point of the process using a MicropH 2002 pH meter equipped with a 52-07 pH electrode (Crison, Barcelona, Spain). The positive and negative controls were, respectively, designated as C+ (FOS) and C− (plain medium), while the carotenoids’ colon fraction samples were named β-carotene, lutein, lycopene, mix and alga and were analysed in triplicate. Afterwards, the samples were stored at −20 °C until analysis. All the procedures within this section were conducted inside an anaerobic workstation from Don Whitley Scientific in West Yorkshire, UK [28,29,30,31,32].

#### 2.2.4. Faecal Fermentation Samples’ Processing

Aliquots of the colon fraction samples were subjected to centrifugation at 4 °C for 10 min at 4000× *g*. The resultant supernatants were used for the assessment of sugar and organic acid content, as detailed in Section 2.3, while the pellet was employed for genomic DNA extraction, following the procedure outlined in Section 2.4 [28,29,30,31,32].

The methodology of faecal fermentations offers several advantages crucial for understanding microbial dynamics and their impact on health. Firstly, it involves collecting fresh faecal samples from diverse healthy donors, ensuring a representative microbial composition. The meticulous preparation of the inoculum, including steps to dilute the faecal matter and maintain anaerobic conditions, enhances reproducibility. Controlled environmental conditions are achieved through anaerobic workstations and specific fermentation media, mimicking the colon’s anaerobic environment. Regular sampling and pH measurements enable detailed monitoring of microbial activity and metabolic changes over time. Additionally, the inclusion of positive and negative controls, alongside triplicate sample analyses, ensures robust and reliable experimental results. Ultimately, this methodology offers valuable insights into the complex microbial community dynamics that are crucial for understanding gut microbiota interactions and their implications for host health [33,34,35,36].

### 2.3. Sugars and SCFAs Analysis

Sugar consumption and organic acid production during faecal fermentation were analysed using an HPLC system. This system consisted of a Knauer K-1001 pump (Berlin, Germany) equipped with an ion exchange Aminex HPX87H (300 × 7.8 mm) (Bio-Rad, Hercules, CA, USA) column and two detectors assembled in series, namely a UV-vis detector (220 nm) and a refractive index detector, both from Knauer (Berlin, Germany). The entire setup was maintained at a temperature of 65 °C. An isocratic gradient was implemented, utilizing a solution of 13 mM H_2_SO_4_ (Merck, Darmstadt, Germany) at a flow rate of 0.6 mL/min. Each injection involved a 40 µL volume, and the total running time for the analysis was set to 30 min. Before injection, fermentation supernatants were filtered through a 0.22 µm syringe filter, and each sample was injected twice for duplicate analysis The identification and quantification were achieved by comparison of the relative retention times of sample peaks with standards and using calibration curves for glucose, acetic, succinic, propionic, butyric and malic acids [28].

### 2.4. Bacterial Population Analysis

#### 2.4.1. DNA Extraction

An NZY Tissue gDNA Isolation kit (NZYTech, Lisbon, Portugal) was used to extract DNA, with slight modifications. Briefly, pellets were washed with TE buffer (pH 8.0; Tris EDTA buffer), vortexed, and centrifuged at 4000× *g* for 10 min at 4 °C. Then, supernatants were discarded and 180 μL of a freshly prepared lysozyme solution (10 mg/mL lysozyme in TE buffer 1×) was added to the samples and incubated for a period of 2 h at 37 °C. Afterwards, samples were centrifuged at 4000× *g* for 10 min at 4 °C and supernatants were discarded. The subsequent steps adhered to the manufacturer’s guidelines. After extraction, the DNA’s purity and concentration were determined using a Thermo Scientific^TM^ μDropTM Plate coupled with a Thermo Scientific^TM^ Multiskan^TM^ FC Microplate Photometer (Thermo Fisher Scientific, Waltham, MA, USA).

#### 2.4.2. Metagenomics Analysis

The analysis of 16S amplicon metagenomics sequencing was executed by Novogene Europe in Cambridge, United Kingdom. Targeting the 16SV45 regions, the amplification of 16S rRNA genes utilized specific primers paired with barcodes (5′- GTGCCAGCMGCCGCGGTAA-3′ and 5′-CCGTCAATTCCTTTGAGTTT-3′). The PCR reactions were conducted using 15 µL of Phusion^®^ High-Fidelity PCR Master Mix from New England Biolabs, with 0.2 µM of forward and reverse primers, and 10 ng of template DNA. The PCR conditions encompassed an initial denaturation at 98 °C for 1 min, followed by 30 cycles of denaturation at 98 °C for 10 s, annealing at 50 °C for 30 s, elongation at 72 °C for 30 s, and a final extension at 72 °C for 5 min. Then, following electrophoresis using a 2% agarose gel, the resultant PCR products underwent purification, employing a Universal DNA Purification kit (DP214, TianGen, Sichuan, China). Following purification, library generation utilizing the NEB Next^®^ Ultra™ II FS DNA PCR-free library Prep Kit (E7430L, New England Biolabs, Ipswich, MA, USA) was conducted, incorporating index codes. Library quality was assessed using Qubit and real-time PCR for quantification, alongside the bioanalyzer which was used for size distribution detection. Quantified libraries were pooled and subjected to sequencing on Illumina platforms. Analyses of amplicon sequence variants (ASVs), species annotation, and construction of phylogenetic relationships were executed using QIIME2 software (version QIIME2-202006). α-diversity analysis encompassed 7 indices within QIIME2 software, Observed_otus, Chao1, Shannon, Simpson, Dominance, Good’s coverage, and Pielou_e, to evaluate community diversity, richness, and uniformity. β-diversity analysis, aimed at assessing community composition complexity and comparing sample group differences, utilized unweighted UniFrac distances in QIIME2.

### 2.5. Metabolomics Analysis

UPLC-qTOF MS analysis was employed to elucidate the compound structures and identify fragmentation patterns formed during the 48 h of fermentation. The carotenoid analysis followed the procedure outlined by Monforte et al. [37], with minor adjustments, and was conducted on an Ultimate 3000 Dionex UHPLC coupled to an ultra-high-resolution Qq-time of flight mass spectrometer (Impact II, Bruker Daltonics, Germany). Metabolite separation used a reversed-phase column (ACQUITY UPLC^®^ BEH 130Å C18, 1.7 μm, 2.1 × 100 mm, Waters, Milford, MA, USA). The mobile phases used were as follows: A (acetonitrile/water (90:10 *v*/*v*)); B (ethyl acetate); flow: 0.250 mL/min; 0–2 min: 15% A; 2–11.6 min: 15 to 0% A; 11.6–14 min: 0 to 15% A; 14–15 min: 15% A. For carotenoid analysis, electrospray ionization (ESI) used MS parameters in the positive mode, covering a mass range from *m*/*z* 50 to 2000 in the auto MS scan mode. Specific settings included a capillary voltage of 4.5 kV, drying gas temperature of 200 °C, a drying gas flow rate of 8.0 L/min, nebulizing gas pressure of 2 bar, collision radio frequency (RF) of 300 Vpp, transfer time of 120 μs, and pre-pulse storage of 8 μs. Following acquisition, internal mass calibration was performed by employing sodium formate clusters delivered by using a syringe pump at the beginning of each chromatographic analysis. Subsequent data analysis was carried out using Metaboscape^®^ software (version 2023).

### 2.6. Statistical Analysis

Statistical analysis of the data was performed using IBM SPSS Statistics v21.0 (IBM, Chicago, IL, USA). The normality of the data distribution was assessed using the Shapiro–Wilk test. Since the data exhibited a normal distribution, the significance of the carotenoids’ impact on bacterial populations at each time point was determined by employing one-way ANOVA, followed by Tukey’s post hoc test. For the assessment of the carotenoid solutions’ effect on the bacterial population over time, repeated measures ANOVA was utilized. Statistical significance was established for *p*-values of ≤0.05.

## 3. Results and Discussion

### 3.1. The Impact of the Digested Carotenoids on Organic Acid Production

The colon’s environmental conditions, particularly its acidity, significantly influence the composition of intestinal microbes, affecting nutrient availability, enzyme function, and microbial development [38]. Moreover, the faecal fermentation medium’s pH serves as a crucial indicator of the fermentation process, with a decrease in acidity associated with the presence of organic compounds and SCFAs [39].

**Figure 1:**
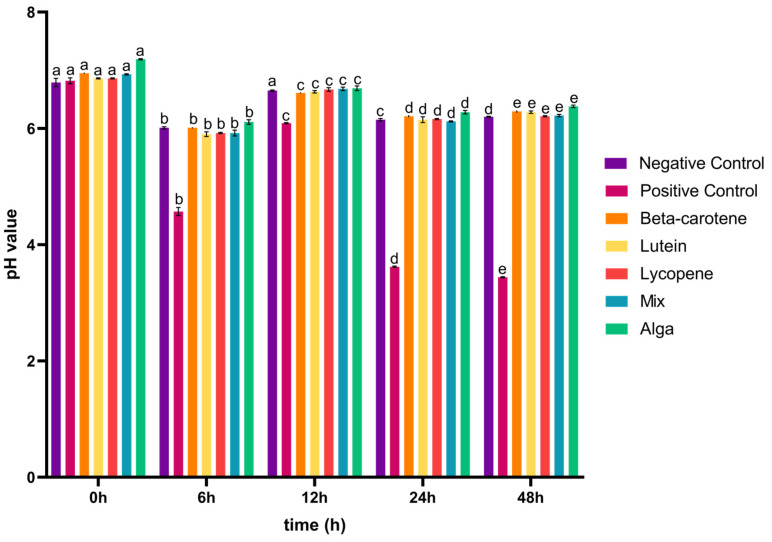
Variation in pH values (mean ± SD) over 48 h of faecal fermentation. Negative control: faecal inoculum without any sample; positive control: faecal inoculum with FOS; carotenoid sample groups: faecal inoculum in the presence of the different carotenoids’ colon fraction samples, obtained after simulated GID. Mix: mixed solution of β-carotene, lutein, and lycopene. Alga: *Osmundea pinnatifida*. Different letters mark statistically significant (*p* < 0.05) differences.

As shown in Figure 1, the pH values exhibited a common trend for both controls and colon fraction samples. There is a decrease in pH from 0–6 h to 12–24 h of incubation, followed by an increase from 6–12 h to 24–48 h, except for the positive control, where the pH slightly decreases between 24 and 48 h. This suggests that the production of organic acids may have occurred during the initial decrease in pH from 0–6 h to 12–24 h of incubation.

**Figure 2:**
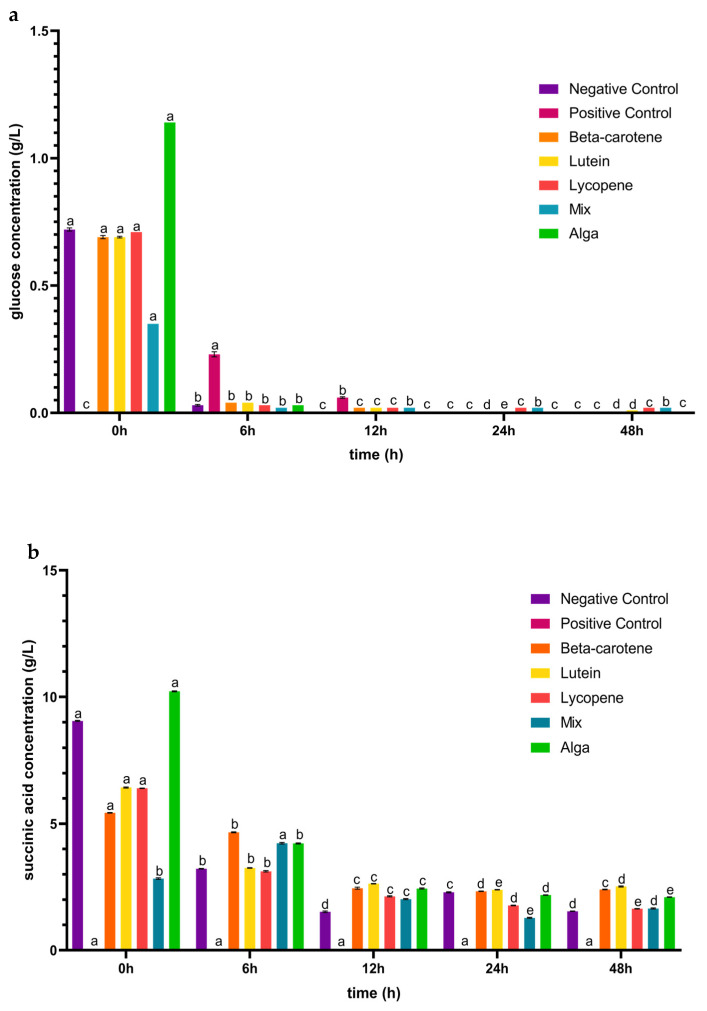

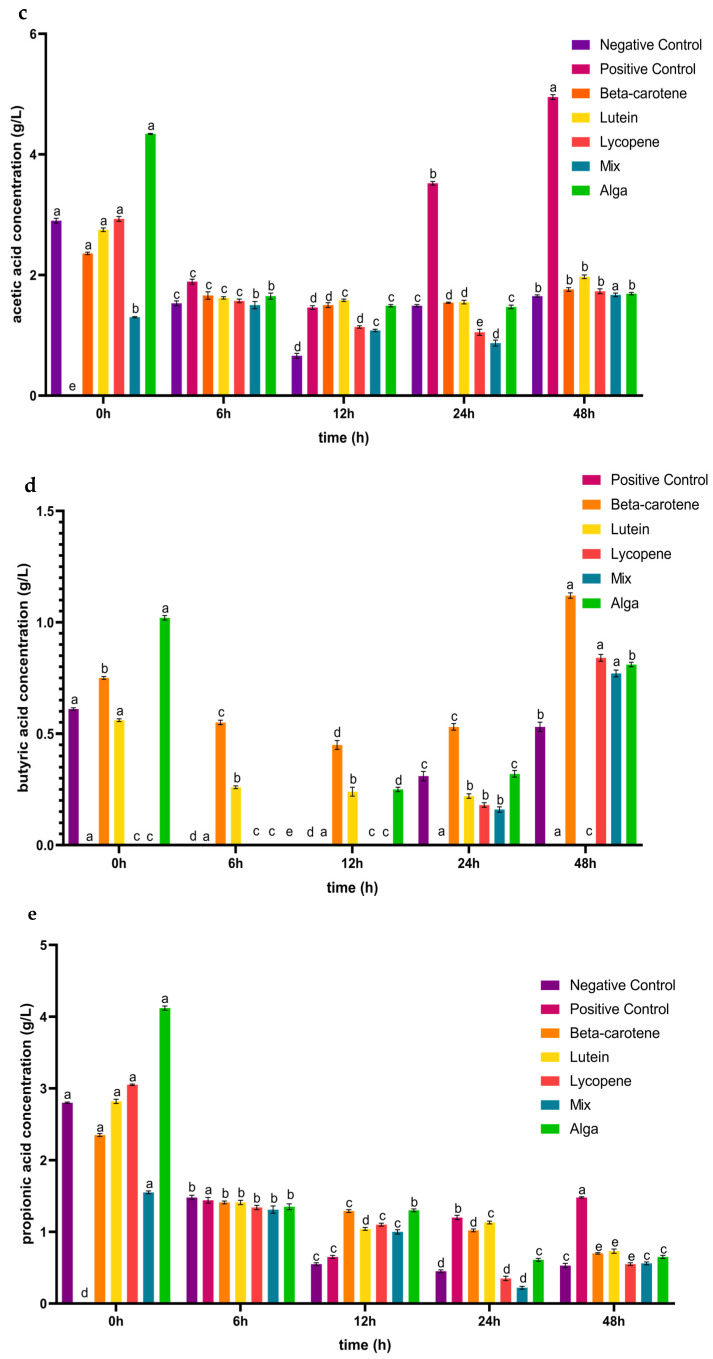

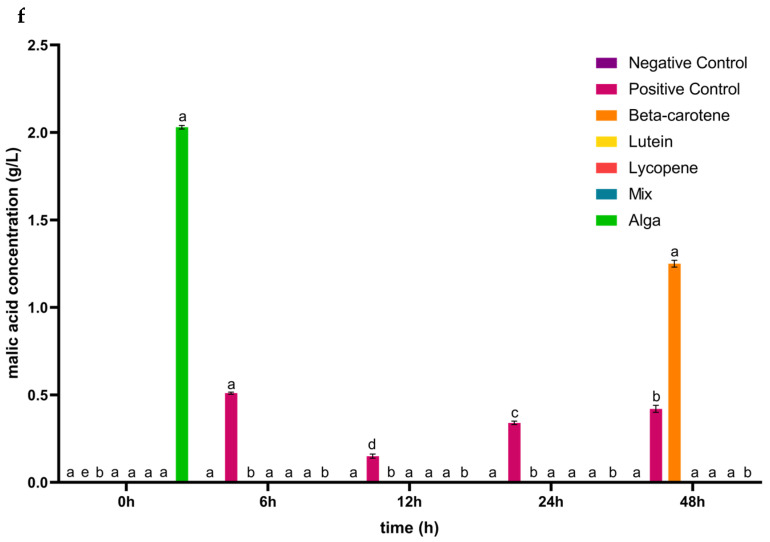
Concentrations (mean ± SD) of glucose consumed (**a**) and of organic acids (**b**–**f**) released, in g/L, during the 48 h of incubation in the presence of the carotenoid sample groups after simulated GID. Mix: mixed solution of β-carotene, lutein, and lycopene. Alga: *Osmundea pinnatifida*. Different letters mark statistically significant (*p* < 0.05) differences.

**Figure 3:**
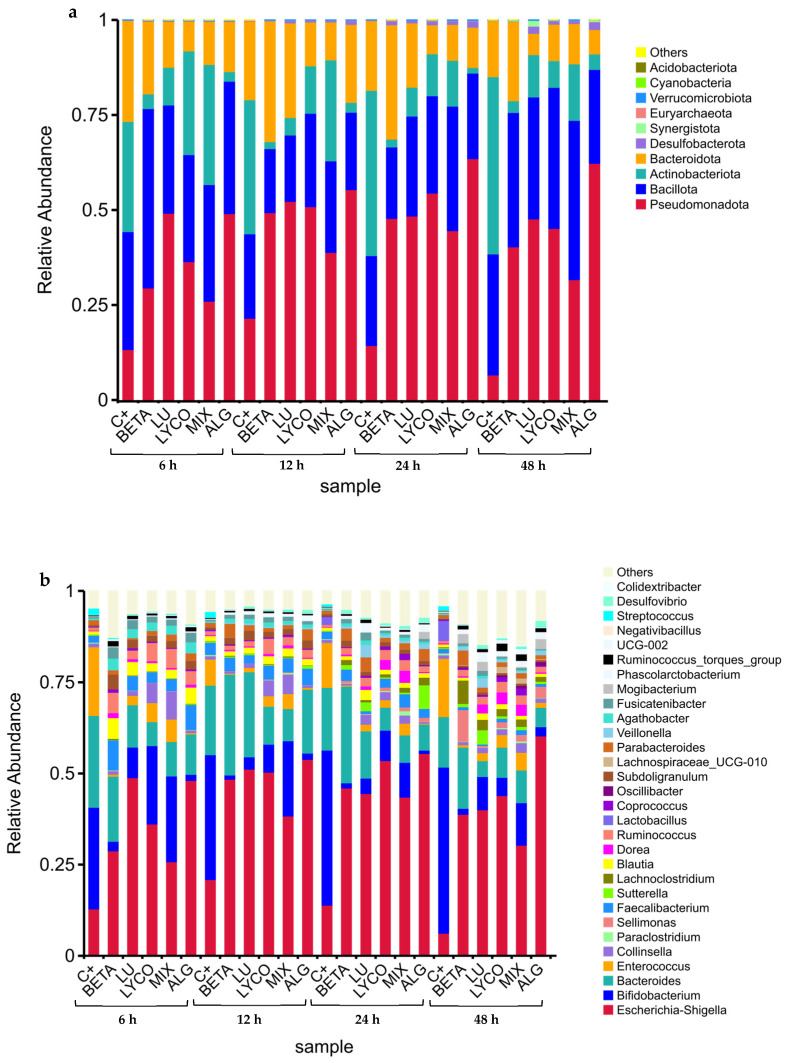
Phyla (**a**) and genera (**b**) relative taxonomic abundances from each tested condition at each time-point. (C+: control; BETA: β-carotene; LU: lutein; LYCO: lycopene; MIX: mixed solution of β-carotene, lutein, and lycopene; ALG: *O. pinnatifida*).

After the simulated GID, the carotenoids’ colon fraction for all the tested conditions were collected and analysed in terms of sugars metabolized and organic acids produced. In Figure 2a, it is possible to observe that glucose was the primary sugar metabolized when exposed to the carotenoid samples. The glucose concentration was initially high due to large amounts of this monosaccharide in the fermentation medium. After 6 h, there was a marked decrease in its concentration in all the conditions, as the bacteria strains consumed this substrate. After 12 h, the glucose concentration was even lower for all the tested conditions, and in the case of the alga group, the monosaccharide was completely consumed. Peculiarly, in the case of the lutein group, an increase in glucose concentration was detected between 24 and 48 h of faecal fermentation. This may be related to an increase in Bacillota phyla during this period, as observed below in Figure 3. This increase could lead to heightened metabolic activity, particularly in using glucose present in the faecal sample. Initially, this may cause a decrease in glucose concentration, but as Bacillota populations grow, they may release byproducts or intermediates that serve as substrates for other microorganisms [40], potentially leading to a subsequent increase in glucose concentration as fermentation progresses beyond 24 h.

Figure 2 also provides insights into the capacity of the IM to generate a range of organic acids, including succinic, acetic, butyric, propionic, and malic acids. For succinic acid (Figure 2b), in contrast to the positive control, the carotenoid samples triggered the production of this acid during the 48 h incubation period following simulated GID. For the β-carotene and lutein sample groups, the concentration of succinic acid initially decreased until the 24 h mark, after which it increased from 24 to 48 h of the process. Lycopene and the alga group exhibited a similar trend, with a decrease in succinic acid concentration over the 48 h incubation period. In the case of the mix sample, the concentration of succinic acid increased in the first 6 h and from 24 to 48 h of incubation and then decreased between 6 and 24 h.

Regarding acetic acid (Figure 2c), and contrary to the positive control, the carotenoid samples induced an early production of this acid (from 0 h), although the concentration of acetic acid was lower than the mentioned control in the following 42 h of the fermentation process. The changes in concentration over the 48 h incubation period followed a similar pattern for the lutein, lycopene, and alga groups, with a decrease until the first 24 h followed by an increase in the subsequent 24 h. For β-carotene, the concentration of acetic acid initially decreased in the first 12 h and then increased for the remainder of the incubation time. In the case of the mix sample, the concentration of acetic acid increased during the 0–6 h and 24–48 h intervals but decreased between 6 and 24 h of fermentation. These findings deviate from a prior study, in which the concentration of acetic acid increased throughout the entire process for plain β-carotene, lutein, and lycopene [41]. This can be linked to the fact that the Bacteroidota phylum generally had a decreased abundance during the fermentation process in the presence of the carotenoid sample groups, as discussed in Section 3.2.

As for butyric acid (Figure 2d), the concentration variation was consistent for lycopene and the mix sample groups, increasing in concentration after 24 h of fermentation. In the case of β-carotene, the concentration of this acid decreased in the first 12 h of the simulated fermentation and increased from 12 h to 48 h of the process, while the lutein sample concentration decreased during the incubation process. The alga sample group displayed a decrease in the first 6 h and an increase from that point until the end of the process. However, unlike the positive control, the carotenoid samples induced the generation of this acid during the 48 h incubation period following the simulated GID. Once again, these observations diverge from prior research, where the concentration of butyric acid increased over the entire 48 h process for plain β-carotene and lycopene. For plain lutein, the concentration increased from 6 to 12 h and decreased from 12 to 24 h [41].

For propionic acid (Figure 2e), the concentration of this acid decreased during the first 24 h of incubation in the plain lycopene, the mix, and the alga samples, increasing from this point until the end 24 h of the simulated fermentation. The plain lutein sample induced a decrease in the concentration of this acid in the first 12 h and the last 24 h of this process, while it increased between the 12 h to 24 h. In the case of plain β-carotene, the propionic acid concentration decreased over all of the simulated fermentation. In a previous study, the concentration of propionic acid increased until the end of the process for β-carotene and lycopene, and for lutein it increased from 6 to 12 h and decreased in the last 12 h of incubation [41]. The production of malic acid (Figure 2f) was only induced in the β-carotene sample during the last 24 h of the process and in the alga sample during the first 6 h of incubation.

In conclusion, although the microbiota pH value decreased (Figure 1) between the 0–6 h and the 12–24 h of incubation, it is not possible to establish a correlation with the production of organic acids (Figure 2b–e) since this occurred during the 48 h of the process with only the concentration of the acids varying along the incubation time. Moreover, the induction of these acids’ production by the tested groups corroborates the fact that Lachnospiraceae, *Lactobacillus*, and *Bifidobacterium* bacteria, which are known for their ability to produce these types of acids [22], were significantly present in the intestinal microbiota under the tested conditions (further explored above in Section 3.2).

### 3.2. Microbiota

#### 3.2.1. Relative Abundance

To assess the effects of different carotenoids on the IM’s population dynamics, fresh faecal samples were evaluated with a technique based on the extraction of DNA and the amplification of the 16S ribosomal RNA gene (rRNA), which can be exploited with polymerase chain reaction (PCR) and metagenomics sequencing.

The main taxa present in each tested condition (control, β-carotene, lutein, lycopene, mix, and alga) at phylum and genus levels were chosen based on the findings of the taxonomic annotation to create a distribution histogram of the relative abundance (RA) of taxa. The relative taxonomic abundances of phyla and genera found in each tested condition during the 48 h of fermentation are illustrated in Figure 3a and Figure 3b, respectively.

In Figure 3, it is possible to observe the RA of some bacterial groups in the IM for each sample condition during the 48 h of the simulated fermentation process.

At the phylum level (Figure 3a), the IM was mainly composed of Bacteroidota, Bacillota, Actinobacteriota, and Pseudomonadota, which is consistent with the results obtained in other studies with the plain carotenoids tested (β-carotene, lutein and lycopene) [41,42].

During the 48 h of the simulated fermentation the RA of Pseudomonadota phylum increased for all tested groups in comparison with the control group, contributing to the homeostasis of the anaerobic environment of the GI tract and, hence, the stability of the strictly anaerobic microbiota [43]. These results align with those obtained in previous studies for the individual carotenoids [41,42], allowing us to conclude that our samples positively impact intestinal health.

In terms of the Bacteroidota phylum, its RA only increased from 12 h to 48 h of simulated fermentation in the plain β-carotene group and at 12 h of this process for the plain lutein group, compared with the control. For the other tested conditions, the RA of this phylum was lower than the control during all fermentation processes, which goes against the results obtained in previous studies for plain lutein and lycopene [41,42]. However, the tested samples did not contain a significant quantity of polysaccharides and oligosaccharides, which are metabolized by this phylum [44], explaining the decrease in the RA of Bacteroidota observed.

For the Bacillota phylum, all the tested groups presented the same behaviour along the 48 h of fermentation, which was opposite to the control. The RA of this phylum decreased from 6 h to 12 h of the process and increased from 12 h to 48 h. In addition, an increase in the RA of this phylum was observed for the plain lycopene and the mix groups between 12 h and 48 h of the fermentation process, and also for the plain lutein group and the plain β-carotene group between 24 h and 48 h and after 48 h of fermentation, respectively. These results are similar to the ones obtained in previous studies for plain carotenoids, evidencing the positive role of these carotenoids in intestinal health. However, a decrease in the RA of the Bacillota phylum was observed for the alga group, which might not necessarily be negative if accompanied by an increase in other beneficial phyla [45]. Also, in opposition to the pure carotenoids’ samples, *O. pinnatifida* has other components such as polyphenols, fibre, and proteins [46] that influence microbiota modulation [47].

The RA of the Actinobacteriota phylum decreased during the 48 h of the simulated fermentation process for all the tested conditions compared with the control group. These results are in line with those obtained previously [41,42] for the plain carotenoids and can be supported by the fact that the tested samples are low in fermentable oligosaccharides, disaccharides and polyols, and fructooligosaccharides and galactooligosaccharides (FODMAPs), leading to a decrease in Actinobacteriota phylum, which is associated with its metabolization [48].

Within the *Bacillota* phylum, 23 distinct genera were identified, as illustrated in Figure 3b. These genera are affiliated with various families, including Lachnospiraceae (*Sellimonas*, *Lachnoclostridium*, *Blautia*, *Dorea*, *Coprococcus*, UCG-010, *Agathobacter*, *Fusicatenibacter*, *Ruminococcus torques* group), Enterococcaceae (*Enterococcus*), Peptostreptococcaceae (*Paraclostridium*), Ruminococcaceae (*Faecalibacterium*, *Ruminococcus*, *Subdoligranulum*, *Negativibacillus*), Lactobacillaceae (*Lactobacillus*), Oscillospiraceae (*Oscilibacter*, UCG-002, *Colidextribacter*), Veillonellaceae (*Veillonella*), Anaerovoracaceae (*Mogibacterium*), Acidaminococcaceae (*Phascolarctobacterium*), and Streptococcaceae (*Streptococcus*).

In the families Lachnospiraceae, Ruminococcaceae, and Oscillospiraceae, the tendency of an increase observed in the RA for all the tested conditions (Figure 3b) during the simulated fermentation process, in comparison with the control. This may indicate that the carotenoid samples favoured the growth of these bacteria, which are associated with overall health and play important roles in the microbial community [49]. However, exceptions were noted for the UCG-010, *Faecalibacterium*, *Negativibacillus*, and *Colidextribacter* genera, where RA decreased when plain lutein was tested.

The Veillonellaceae, Anaerovoracaceae, and Acidaminococcaceae families consistently exhibited higher RA than the control in all conditions between the 12 h and 48 h of the fermentation process, which indicates that the samples promoted the growth of these bacteria. Conversely, the genera *Enterococcus*, *Lactobacillus*, *Streptococcus*, and *Paraclostridium* showed a decrease in RA across all carotenoid groups, except for the mix sample, where RA specifically increased for the *Paraclostridium* genus between the 12 h and 24 h of the simulated fermentation compared to the control.

Regarding the Actinobacteriota phylum (Figure 3b), the RA of *Bifidobacterium* and *Collinsella* genera were lower than those reported for the control in all conditions tested. However, for the lutein, lycopene and mix groups, the RA of the *Collinsella* genus was higher than that observed for the control.

Within the Bacteroidota phylum (Figure 3b), the presence of *Bacteroides* and *Parabacteroides* genera was detected. In the *Bacteroides* genus, a reduction in relative abundance (RA) was noted across all the sample conditions compared to the control, except for the plain β-carotene sample, where a minor increase in its respective RA was observed between the 12 h to 48 h. Conversely, for the *Parabacteroides* genus, the RA increased in all sample conditions compared to the control. This suggests that carotenoids may create favourable conditions for the proliferation of bacteria belonging to this genus.

In the Pseudomonadota phyla (Figure 3b), the *Escherichia*-*Shigella* and *Sutterella* genera were detected in the IM in the presence of the tested conditions at a slightly higher RA when compared with the control. This increase in the RA of these bacteria may suggest a state of imbalance in the microbial community (microbial dysbiosis) of the IM, which can have implications for gut health and function [50,51].

In a general way, the tested carotenoid samples promoted the growth of beneficial phyla that are associated with the production of SCFAs, which supply energy to colonic cells, supporting their regeneration and contributing to the maintenance of optimal intestinal barrier impermeability [52]. However, they also promoted the growth of bacteria such as *Escherichia*, *Shigella*, and *Sutterella*, which are related to negative effects such as dysbiosis [53,54]. In future work, different concentrations of carotenoids solutions should be tested to assess the possible dose effect on the growth of beneficial or/and prejudicial bacteria in IM.

#### 3.2.2. Compositional Analysis

**Figure 4:**
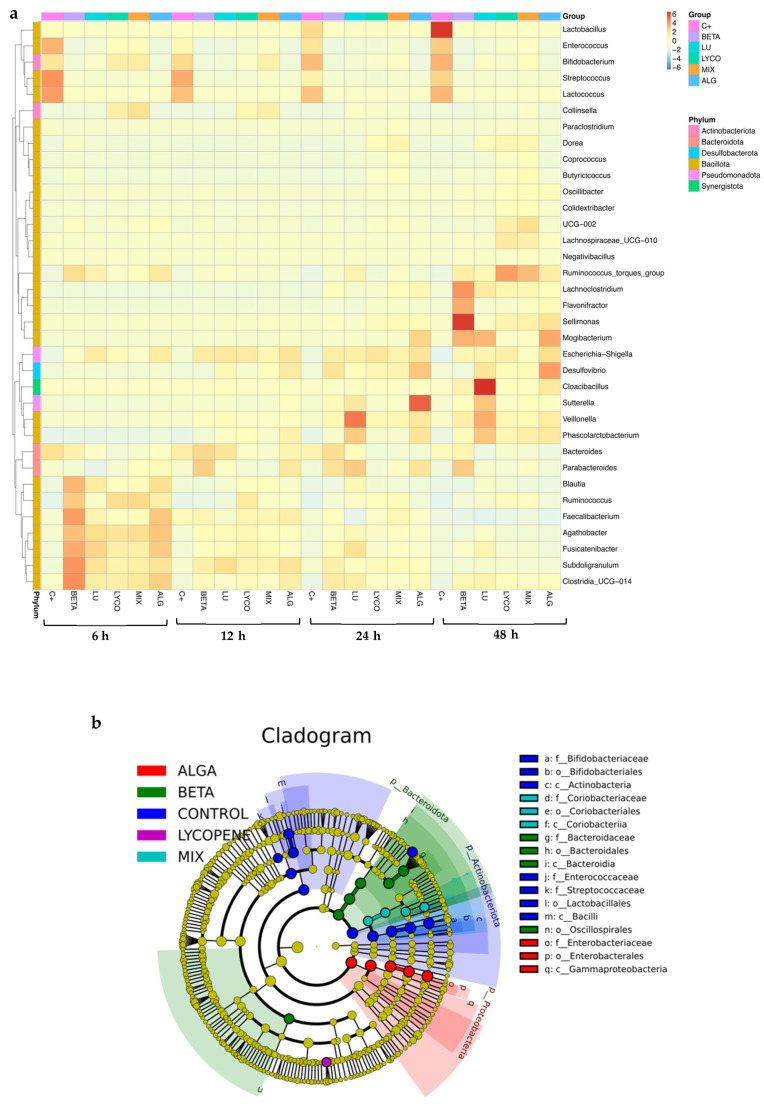
Composition analysis of IM in each tested condition. (**a**): Taxonomic abundance cluster heatmap. (**b**): Graphical representation of phylogenetic relationships—cladogram. (C+: control; BETA: β-carotene; LU: lutein; LYCO: lycopene; MIX: mixed solution of β-carotene, lutein, and lycopene; ALG: *O. pinnatifida*).

A heatmap (Figure 4a) was created based on the microbial abundance data of the primary genera present in all the sample conditions to determine whether or not samples that have undergone comparable processing are grouped and to identify potential similarities and differences between groups. Furthermore, the cladogram shown in Figure 4b offers insightful information on the evolutionary relationships among the various studied groups of microorganisms based on their genetic similarity and shared evolutionary background.

Figure 4a illustrates the most abundant genus and phylum in each sample throughout the 48 h of the simulated fermentation. For plain β-carotene, the most abundant phylum was Bacillota. However, when compared to the control, notable differences were observed. At 6 h of fermentation, there was an increase in bacteria belonging to the *Faecalibacterium*, *Subdoligranulum*, and *Clostridia* genera within the Bacillota phylum. After 48 h, bacteria such as *Sellimonas* and *Lachnoclostridium* became more prominent. These findings are consistent with previous studies [42] and support the beneficial impact of β-carotene on intestinal health.

In the case of plain lutein, the most abundant phylum continued to be Bacillota, as in the control and β-carotene groups, but it also presented an abundance of the Bacteroidota phylum. Nevertheless, there was an increase in the Lachnospiraceae family and a decrease in *Lactobacillus*, *Enterococcus*, *Streptococcus*, and *Bifidobacterium* bacteria, which corroborates previous results that sustain the beneficial roles of lutein in intestinal well-being [41].

In the plain lycopene and mix samples, the Bacillota phylum remained dominant in terms of abundance. Specifically, in the lycopene sample, the most prevalent genus was *Ruminococcus* (from the Bacillota phylum), while in the mix sample, it was *Collinsella* (from the Actinobacteriota phylum). The alga sample exhibited more noticeable differences compared to the control, as the most abundant genera belonged to different phyla, namely Desulfobacteriota, Bacillota, and Pseudomonadota.

In summary, the observed increase in the Bacteroidota and Bacillota phyla suggests that carotenoids may indeed contribute to regulating intestinal health.

It is feasible to see the evolutionary connections between each group in Figure 4b. The circles extending from the inner side to the outer side symbolize the taxonomic rank from phylum to genus, and each circle represents a distinct taxon at the corresponding taxonomic level. The diameter of each circle represents the proportionate abundance of each taxon. Taxa that differ significantly are shown by the colour of the corresponding group, whereas taxa that differ less significantly are indicated by the colour yellow. Thus, by analysing Figure 4b, it is easy to confirm that Bacteroidota, Pseudomonadota, and Actinomycetota are the three most prevalent phyla.

For plain β-carotene, in comparison with the other conditions, the most abundant phylum is Bacteroidota, but the Oscillopirales order was also significant in this condition. In the case of plain lutein, it is absent from Figure 4b, which indicates that there are no species with significant differences in this condition.

Since lycopene’s representation is not found in the circles that correlate to the phyla with the highest relative abundance, it has a phylogenetic relationship more distant from the other tested groups. However, by analysing the heatmap (Figure 4a), it is possible to observe that the phylum represented in the cladogram (Figure 4b) may correspond to Bacillota since it is the phylum with the highest relative abundance in this group.

In Figure 4b, is also possible to observe that Actinobacteriota is the most significant phylum in the mix condition and that the Pseudomonadota phylum is most significant in the alga condition, in comparison with the other tested conditions.

Therefore, it is possible to conclude that these results corroborate the ones previously analysed in Figure 3 and Figure 4a.

#### 3.2.3. Flower Diagram

**Figure 5:**
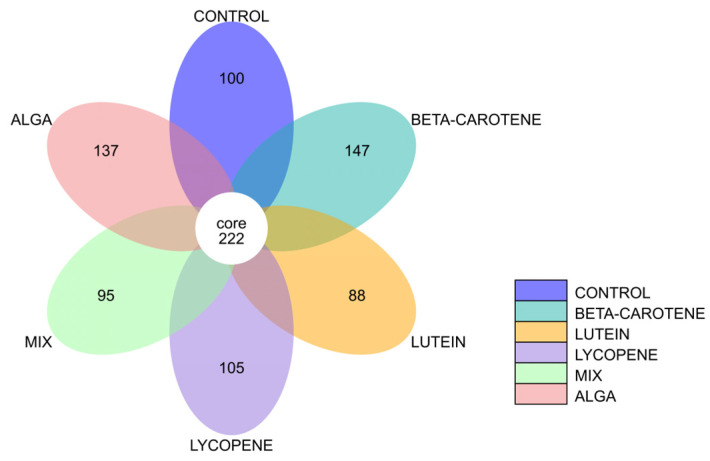
Flower diagram of each sample group. MIX: mixed solution of β-carotene, lutein, and lycopene. Alga: *O. pinnatifida*.

Below, Figure 5 presents the flower diagram of each sample group. In this diagram, each petal represents an individual group sample, and distinct colours are used to distinguish between different groups. The central core number signifies the count of feature sequences that are common to all groups, while the numbers on each petal indicate the number of unique feature sequences specific to each group.

As a result, one can observe that the sample groups share a total of 222 feature sequences among them. Notably, the plain β-carotene and algae conditions stand out, with the highest number of unique feature sequences. This finding suggests that these two groups harbour a greater diversity of unique bacterial species, signifying higher bacterial diversity when compared to the other tested conditions.

#### 3.2.4. Ternary Plot

**Figure 6:**
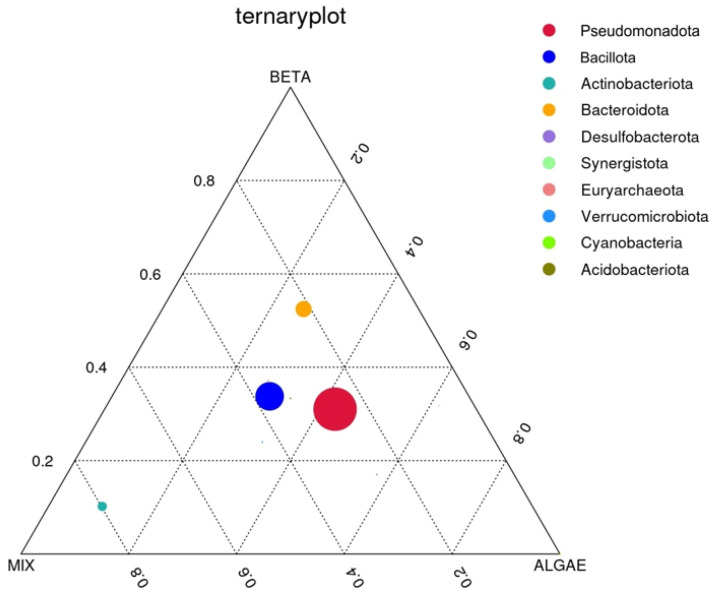
Ternary plot of the plain β-carotene (BETA), mix (mixed solution of β-carotene, lutein, and lycopene), and alga (*O. pinnatifida*) assay conditions.

To identify distinctions in the predominant taxa within the three sample groups at the phylum level, we opted to create a ternary plot using the top 10 taxa, each with the average abundance across all three sample groups. This ternary plot, specific to the phylum level, is represented in Figure 6 below:

The three vertices in the plot correspond to the three distinct groups: β-carotene, mix, and alga. Within the plot, the circles symbolize the predominant taxa, with their sizes reflecting their relative abundances. A circle’s proximity to a specific sample group signifies a higher abundance of that particular phylum in that group.

In Figure 6, it is observed that the prevailing taxa are the Pseudomonadota, Bacillota, Bacteroidota, and Actinobacteriota phyla, listed in decreasing order of relative abundance. The circle representing the Actinobacteriota phyla is notably closer to the mix group, indicating a higher abundance of this phyla in comparison to the other two groups. While the other three circles are positioned more centrally on the ternary plot, it is discernible that the Bacteroidota phylum exhibits a somewhat higher abundance in the plain β-carotene sample, the Pseudomonadota phylum is to some extent more abundant in the alga sample, and, finally, the Bacillota phylum’s abundance is to some extent higher in the mix sample.

#### 3.2.5. α-Diversity Analysis

The α-diversity was applied to the analysis of the microbial community diversity within a sample [55], in which Shannon and Simpson indexes were calculated based on Operational Taxonomic Unit (OTU) species and abundance, allowing for the analysis of the species richness and evenness [56].

The Shannon diversity index is calculated by considering the total number of taxa in a sample (richness) and the proportion of each taxon (abundance). On the other hand, the Simpson index characterizes the diversity and uniformity of species distribution within a community by calculating the probability that two randomly sampled individuals belong to different species. Therefore, the higher the Shannon index and the lower the Simpson index, the greater the variety of species in the sample.

**Figure 7:**
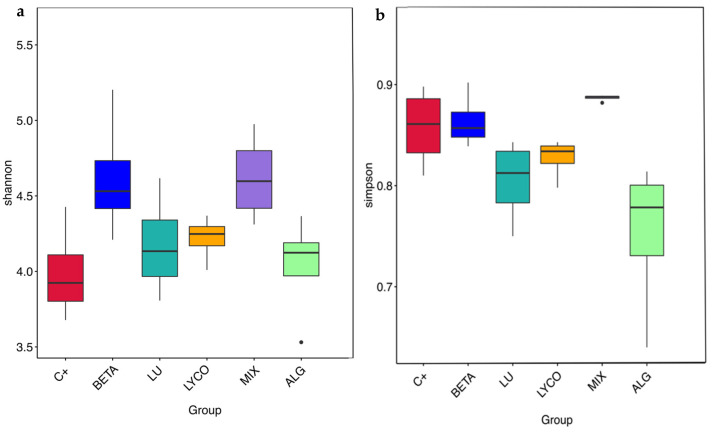
Panels (**a**,**b**) show the distribution of the Shannon and Simpson diversity indices, respectively, across the different tested groups. Each box plot represents the interquartile range (IQR), with the horizontal line inside the box indicating the median value. The whiskers extend to the minimum and maximum values within 1.5 times the IQR from the lower and upper quartiles. Points outside the whiskers represent outliers, indicating data points that are significantly different from the majority of the data. Significant differences were obtained (*p* < 0.05). C+: control; BETA: β-carotene; LU: lutein; LYCO: lycopene; MIX: mixed solution of β-carotene, lutein, and lycopene; ALG: *O. pinnatifida*.

Boxplots were constructed to assess variations in the α-diversity indices across different groups, as illustrated in Figure 7. These boxplots provide a visual representation of key statistical parameters for each experimental condition, including the maximum, minimum, median, and outlier values for the indices.

In Figure 7a, it is observed that the mix and plain β-carotene samples exhibit the highest median values for the Shannon index, while the alga and lutein samples display the lowest Shannon index values. This indicates that the mix and plain β-carotene samples induce a greater diversity of species within the IM compared to the alga and lutein samples. The results obtained for the plain β-carotene sample are not consistent with those obtained in a previous study [41], in which lutein, as a xanthophyll, more significantly impacted the IM than carotenes such as β-carotene. This result shows that carotenoids are structurally different from each other and may alter the composition of the IM in distinct ways [57].

Furthermore, when comparing the median values of the Simpson index (Figure 7b), it becomes evident that the Simpson index for the alga samples is lower than that of the lutein samples. This suggests that the alga samples boast a richer variety of species. Additionally, Figure 7b reveals that the Simpson index for the plain β-carotene samples is lower than that for the mix samples, pointing to a greater species diversity within the β-carotene samples. This observation aligns with the findings in the flower diagram presented in Figure 5.

#### 3.2.6. β-Diversity Analysis

The β-diversity involves comparing the composition of microbial communities in different samples. Initially, this process combines feature sequence information with taxonomy annotation results and feature sequence abundance data to create a table of species abundance. Simultaneously, the phylogenetic relationships between feature sequences are utilized to compute the unweighted Unifrac distance [58,59].

Unifrac distance calculates the distance between samples by using the evolutionary information between microbial sequences in each sample. If there are more than two samples, a distance matrix can be obtained. Then, the weighted Unifrac distance (unweighted Unifrac) is further constructed using the abundance information of the feature sequence [59]. These distances are widely employed as phylogenetic metrics in microbial community sequencing projects.

**Figure 8:**
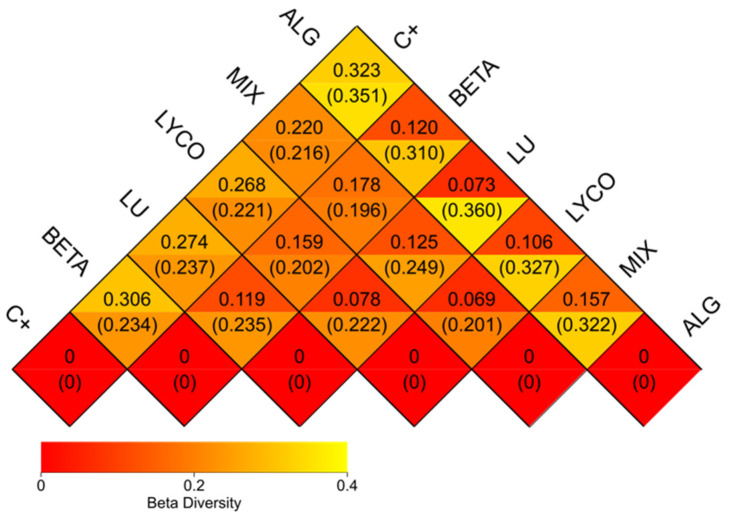
β-diversity heatmap. Note: each grid represents a pairwise dissimilarity coefficient between pairwise samples, in which the weighted Unifrac distance is displayed above and the unweighted Unifrac distance conversely. C+: control; BETA: β-carotene; LU: lutein; LYCO: lycopene; MIX: mixed solution of β-carotene, lutein, and lycopene; ALG: *O. pinnatifida*.

In Figure 8, a heatmap based on the weighted Unifrac and unweighted Unifrac distances is presented, as follows:

The analysis in Figure 8 provides general support for the information previously discussed. Notably, the lower coefficients (0.069) obtained for the plain lycopene and the mix samples suggest a similarity between these two samples at the microbial community composition level, indicating that they share a considerable resemblance.

Conversely, higher coefficients are observed when comparing the control group to each of the other experimental groups. This observation reaffirms that all the samples significantly differ from the control group.

Furthermore, it can be inferred that the microbial community composition of the plain β-carotene sample varies from that of the plain lycopene and the mix samples, as indicated by coefficients of 0.159 and 0.178, respectively, and as previously demonstrated. Additionally, the mix sample exhibits notable dissimilarity from the alga sample, reflecting a coefficient of 0.157.

### 3.3. Metabolomics

#### Principal Component Analysis (PCA)

During this research, each sample (6 h, 12 h, 24 h, 48 h) of each carotenoid group (β-carotene, lutein, lycopene, mix and alga) underwent a detailed analysis using UPLC-qTOF MS. In this study, principal component analysis (PCA) was employed as a powerful statistical method to analyse the UPLC-qTOF MS data obtained from the detailed analysis of carotenoid groups. This analytical approach aimed to compare the compounds’ fragment ions that were potentially generated by the microorganisms present in the IM during the 48 h simulated faecal fermentation process. Each analysis aimed to compare the variance of all compounds’ fragment ions generated in each group along the 48 h process (were obtained for each time-point more than 1000 fragment ions).

By using PCA, we were able to identify major variation trends within the time points for each group, operating on a matrix format where samples corresponded to mass spectra and variables represented individual *m*/*z* ratios of the fragment ions obtained, which means that each variable represented the intensity of signals detected at specific mass-to-charge ratios in the mass spectrometry experiment of the carotenoids group. The number of variables used corresponds to the total number of distinct *m*/*z* ratios detected in the mass spectrometry data for the carotenoid groups.

The PCA computed axis rotations to generate principal components (PCs), aligning with maximal directions of variance in the data, thereby facilitating the interpretation of complex mass spectra [60,61]. Through the computation of scores, loadings, and residual matrices, PCA enabled the visualization of sample differences and disparities in mass spectra and fragmentation patterns [62]. Ultimately, PCA provided insights into the dynamic interactions between carotenoid compounds and the IM, shedding light on the key fragment peak variations along the 48 h and facilitating the identification of relationships between different sample sets [61,63,64,65].

Furthermore, the PCA loading plots that show features related to the observed clustering patterns in order to help in the identification of the variables influencing these patterns were included and are represented below [66].

**Figure 9:**
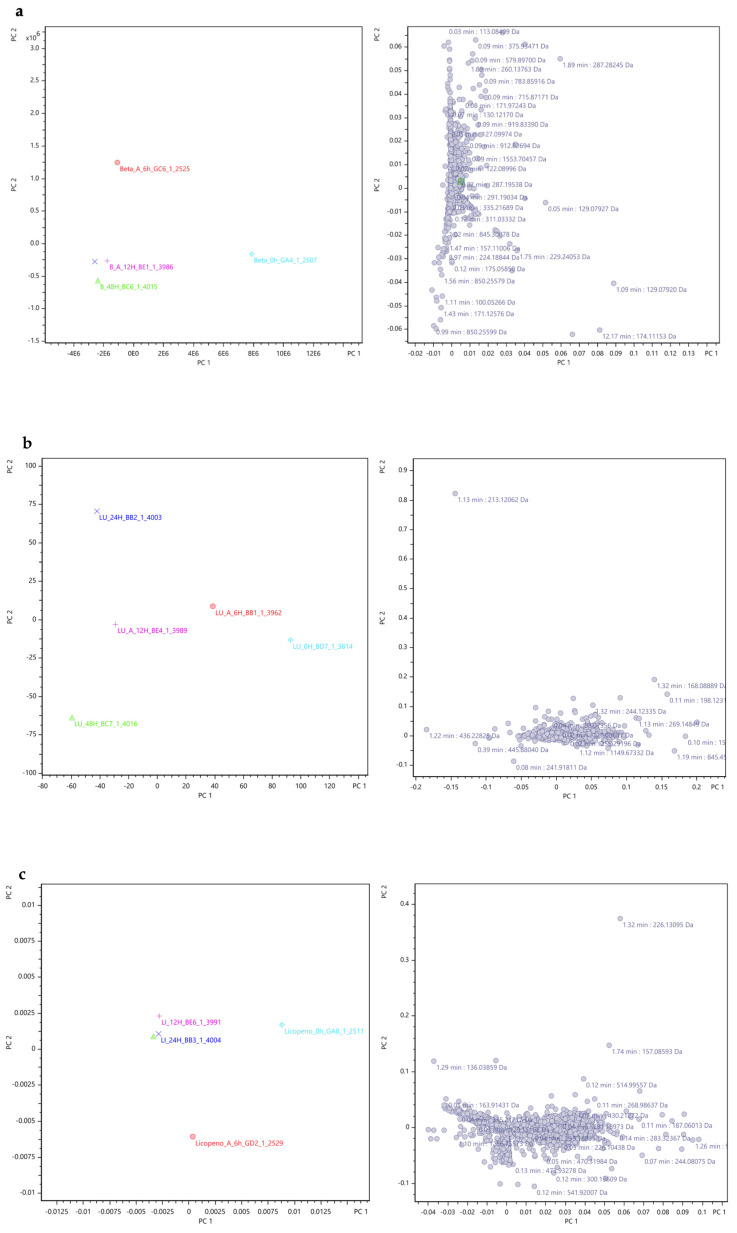

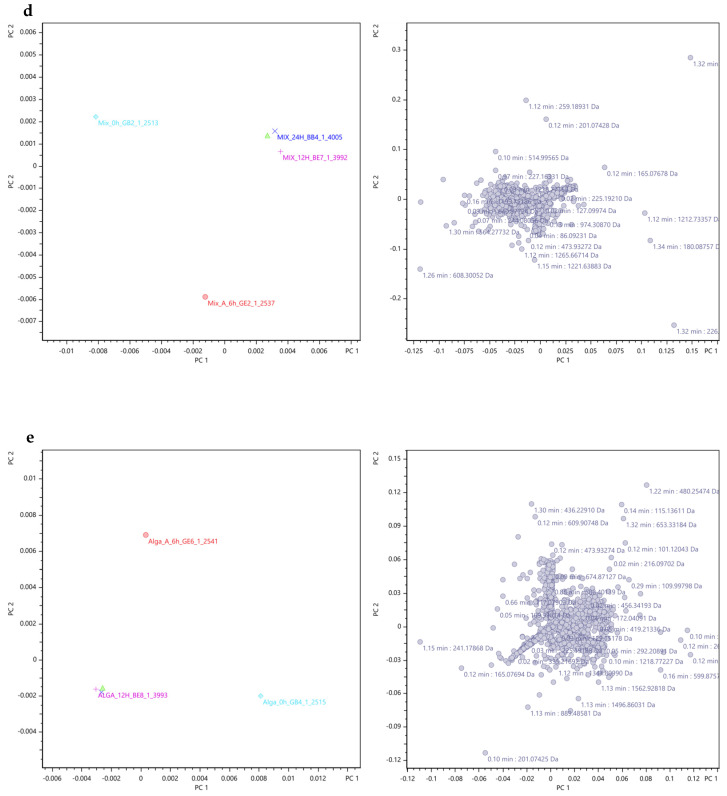
PCA results for the compounds’ fragment ions of the carotenoids’ sample groups along the 48 h of fermentation obtained through Metaboscape^®^ software with Pareto as scaling algorithm. Colours: light blue diamond shape—0 h; red circle—6 h; Pink plus sign—12 h; dark blue cross—24 h; green triangle—48 h. (Beta: β-carotene; LU: lutein; LI: lycopene; MIX: mix; Alga: *O. pinnatifida*). Different shapes represent different time points. Explained variance values: β-carotene (PC1: 66.3% and PC2: 20.7%), lutein (PC1: 53.1% and PC2: 27.0%), lycopene (PC1: 68.1% and PC2: 20.2%), mix (mixed solution of β-carotene, lutein, and lycopene: PC1: 64.7% and PC2: 19.6%), and alga (*O. pinnatifida*: PC1: 54.5% and PC2: 29.6%).

Therefore, the PCA analysis and the PCA loading plots are depicted in Figure 9, wherein distinct graphs represent the results for the plain β-carotene (Figure 9a), plain lutein (Figure 9b), plain lycopene (Figure 9c), mix (mix, Figure 9d), and alga samples (Figure 9e). Also, the explained variance values were indicated to provide a context for interpreting the results of the PCA analysis and to highlight the importance of each principal component in summarizing the data. A higher explained variance value indicates that the principal component explains a larger proportion of the total variability present in the data.

For the samples of plain β-carotene (Figure 9a), plain lycopene (Figure 9c), mix (Figure 9d), and alga (Figure 9e), it can be inferred that the variations in mass ion fragmentation followed a similar pattern over the 48 h fermentation period. Initially, during the early stages of fermentation (at 0 h and 6 h), the peak variances were notably distinct from each other and from the fragments observed in the later time points (12 h, 24 h, and 48 h), exhibiting significant deviation in the graphical representation.

This deviation suggests a marked dissimilarity in the origin of the fragment ions during the initial time points. Conversely, at 12 h, 24 h, and 48 h of fermentation, the variance in fragment peaks became consistently similar, indicating potential relationships between the fragments at these specific time points. However, in the case of plain lutein (Figure 9b), the graph reveals a more scattered distribution of fragment ion peak variances, signifying distinct fragments at each time point. Consequently, it can be concluded that the fragments obtained at different time points exhibit a close association, except for lutein. The similarity among the fragment ions occurs specifically at 12 h, 24 h, and 48 h of fermentation, while the fragment ions obtained at 0 h and 6 h differ significantly from each other and from the fragments observed in the later time points.

The right plots in each Figure 9a–e are loading plots derived from PCA. These plots visualise how different variables contribute to the PCA’s principal components (PC1 and PC2). The purpose of these loading plots is to identify which variables (e.g., mass/charge ratios, retention times) are most influential in defining the principal components and, consequently, the separation and clustering observed in the left PCA scatter plots. Each point on these right plots represents a variable and is annotated with specific values, such as retention times and mass/charge ratios, indicating their contribution to the principal components. The x-axis (PC1) and y-axis (PC2) represent the loadings of the variables on the first and second principal components, respectively. The position of each point indicates how strongly each variable is correlated with these components. 

In these Figure 9a–e, the right plot shows the contribution of variables from the dataset to the principal components, with each point annotated with retention time (min) and mass/charge ratio (Da), highlighting their importance in defining PC1 and PC2. 

Overall, these right plots provide a detailed view of the underlying structure of the data by identifying the variables that drive the principal components, thus offering insights into the most influential factors in the datasets.

## 4. Conclusions

In conclusion, this research provides valuable insights into the dynamic interactions between carotenoids and the IM during faecal fermentation. The environmental conditions of the colon, particularly pH, play a crucial role in shaping the composition of intestinal microbes, affecting nutrient availability, enzyme function, and microbial development. The observed trend of decreasing pH during the fermentation process suggests the production of organic acids, highlighting the metabolic activity of the gut microbiota.

Furthermore, this study demonstrates the capacity of the IM to metabolize glucose as the primary sugar substrate. The production of organic acids, including succinic, acetic, butyric, propionic, and malic acids, varied among carotenoid groups, indicating the influence of carotenoids on microbial metabolic pathways. These findings underscore the potential impact of carotenoids on the production of beneficial SCFAs, such as butyrate, known for their positive effects on metabolic health.

Analysis of the microbial composition at various taxonomic levels revealed that the carotenoid sample groups positively influenced the abundance of beneficial bacterial groups like Lachnospiraceae, Ruminococcaceae, and Oscillospiraceae. However, variations in the abundances of specific genera indicate that the effects of carotenoids on the microbiota are complex and nuanced. Visualization techniques like ternary plots and flower diagrams illustrate the diversity and unique features of the bacterial species in the different sample groups. The analysis of α- and β-diversity confirmed significant differences between the control group and carotenoid groups, highlighting the impact of carotenoids on microbial community structure.

The analysis of carotenoid groups using UPLC-qTOF MS, coupled with PCA, revealed major variation trends in the fragment ions generated during a simulated faecal fermentation process. PCA facilitated the identification of relationships between fragment peaks over the 48 h fermentation period, indicating the distinct origins of fragments at initial time points and closer associations at later time points, except for lutein, which exhibited a scattered distribution of fragment ion peak variances.

In summary, this study underscores the intricate relationship between carotenoids, the intestinal microbiota, and associated metabolites. The positive influence of carotenoids on the abundance of beneficial bacteria and the production of SCFAs suggests potential health benefits associated with carotenoid-rich diets. However, this study also highlights the need for a careful consideration of carotenoid dosages to avoid potential undesired effects on microbial composition. Future research should focus on exploring the long-term effects and potential health implications of carotenoid–microbiota interactions through sustained dietary interventions. This could provide further insights into the role of carotenoids in promoting gut health and overall well-being.

## Figures and Tables

**Table 1:** Identification and quantification (mg/L) of carotenoids in *Osmundea pinnatifida*.

Carotenoids Identified	Concentration (Mean ± SD (mg/L))
Zeaxanthin	33.97 ± 2.49
β-cryptoxanthin	21.14 ± 1.41
Lycopene	33.17 ± 1.80
β-carotene	5.20 ± 0.28

## Data Availability

The original contributions presented in the study are included in the article, further inquiries can be directed to the corresponding author.
